# Thoughts on Success in Dental Practice

**Published:** 1872-08

**Authors:** W. E. Driscoll


### Thoughts on Success in Dental Practice.

BY DR. W. E. DRISCOLL.

*Read Before the Indiana State Dental Society.*

Having recently visited considerably among the members of our profession, I was strongly impressed with the diversity of success that seems to attend their efforts.

All, unquestionably, covet success; and a few thoughts, however crude, bearing upon the subject, can not fail to excite some interest in the minds of the profession, more generally perhaps than any other subject that might be selected. Had some of our members, whose acquirements better qualify them for leading our thoughts on this and other subjects, chosen to do so, you would have found it a subject well worth your attention, and in the proportion that the subject suffers in my hands they must blame themselves for their neglect of it.

To make sure of one merit, at least, and the only one which I could be certain of attaining, I have determined on one which is often a merit—brevity; of which, on the present occasion, I hope you will take due note.

Supposing that all earnestly desire success and dread failure, we may reasonably conclude that if we clearly define the essentials of success, that it will be as sound seed sown on good ground, resulting in an increase upon the former measure of success. It will also devolve upon us to seek out the causes of failure, the weeds that may overshadow and destroy the fairest conditions and prospects, if not uprooted and cast out.

It is not necessary to take time to show that there is a sufficient cause from which springs every success or failure. No one supposes there is no reason for these differences in our fortunes. This being admitted, then, that these results are produced by conditions that may be modified or reversed, and consequently the result be also changed, is it not incumbent upon us to endeavor to understand these reasons, and being forewarned be armed for the contest that is to decide our welfare through the rest of our lives?

Unfortunately for us, plainly as we can see others' mistakes it is hard to admit that *we* have been mistaken and traveling in the wrong direction for the goal of success ourselves. But it will do us good to be told of it, and conviction and reform can be waited for. But what are the essentials of success in dental practice?

Natural fitness, I will venture to affirm, is more imperatively called for in our profession than in any other. And I say so, after more reflection, than on any other point to which I will call your attention.

And there lies ,I believe, the main distinction due us as a profession. We do a work, supply a want better than all others in the world can. Hence, our real and just distinction.

"What can I do best?" is a question of the greatest consequence that a person is ever called upon to decide. For upon that decision the value of a life depends, for it is what one accomplishes that determines his worth. One who performs no labor, or attempts that for which he is naturally unfitted, which is very nearly equal in results, profits no one. Let every one embrace that calling, the duties of which he can perform better than any other, and which gives him at the same time the most pleasure in its pursuit.

What is it that makes our Atkinsons, Tafts, Judds, *et al.* the men of success they are? Not accident. Not because they are so wise they can see through every difficulty that presents itself in practice. But because they have a natural fitness for their profession, which includes, with manipulative ability, a genuine enthusiasm in the work—a love for it. Nothing else could enable them to lead the profession in its great improvements, or maintain them in their eminence. The pecuniary considerations could not, for, no doubt, if they liked some other profession as well, they could reap a far richer reward for their labor.

If, upon learning something of this profession, you do not like it, you should abandon it without delay. No matter how far you may have gone, change to something you can put your heart into, not for your own good alone, but for that of your patients. No one should trust you. It is something too difficult of performance for one who feels no adequate spirit in the work. He will slight it. He may feel himself too good to stoop to the dull details that go to make up a perfect operation. I have known those who felt so. All can understand what kind of dentists they made. The trouble was the other way; the profession of dentistry was entirely too good for them.

The enthusiasm that some feel in their profession is remarkable, but who will say that it is not one of God's greatest blessings? What is ever done so well without as with it? When you lose all animation in the work of our profession, and in all the other means of diverting your thoughts from yourself exclusively, it will be a sad day for you. Happier if you died ere you see that day.

After the ability to master, and a love for the duties of the dentist, must come that thorough training without which no natural capacity can bring anything but a blundering failure in an attempt to perform the difficult and exact duties of the profession. It is not honest in any one to pretend a proficiency in a profession that he knows he does not possess. He might as well—I might say had far better—swindle the people in any of the other modes to which other rogues resort.

One without proper qualifications may persuade himself for a while that it is going to make little difference; but not long; if not utterly obdurate at the outset. Cases of suffering will come to him for relief, which too ignorant to relieve, his conscience will harrass him until he repents of his deception or plunges still further into error, until all have learned to rate him at his just value; then honorable success can never come.

One naturally capable, prefers his business above all others, and has had all the training necessary, may fail of success as a dentist, and for a number of reasons—among them is indolence. It is the ruin of untold numbers in all professions. There is no excellence without labor. Labor is enobling. Whatsoever thy hand findeth to do, do it with thy might; and much more to the point has become proverbs. Yet men in all the professions often fail from this cause, and we do not suspect it.

The physical powers of the idle man becomes enervated; he converts himself into a living sepulchre, loathing himself and all around him. Indolence is opposed by both pagan and Christian philosophy, and common sense. Man was made for action—let him see well to it that he does not thwart the design of his Creator. We all go around a lazy man if we want anything done. We dislike to trouble him. It is painful to see him go to so much trouble to divine some way how not to do it.

It is amazing what industry alone will do without any special talent or advantage. We are astonished at the volumes that some men have written. But industry did it.

Buffon, the distinguished writer on natural history, believed genius to be the result of profound attention directed to a particular subject. Though thoroughly wearied out in the first composition of his works, he compelled himself to go over and improve them, until he found pleasure, instead of weariness, in his long and laborious corrections.

With industry the very odds and ends of time may be worked up into results of the greatest value. An hour each day, withdrawn from some frivolous pursuit, would, if profitably employed, enable a person of ordinary capacity to go far towards mastering a science. It would make an ignorant man well informed in ten years. Time should not be allowed to pass without yielding fruits in the form of something cultivated or some good habit strengthened.

Elihu Burritt owes to the careful employment of his odd moments his wonderful acquirements.

The best works on almost all subjects are the result of thoughts noted, one at a time, through a period taken up more or less with other pursuits, thus preventing their escape into the region of forgetfulness. Newton wrote his chronology, fifteen times before he was satisfied with it, and Gibbon wrote out his Memoir nine times. Sir Walter Scott said there was no necessary connection between genius and an aversion or contempt for the common duties of life.

On the contrary he was of the opinion that to spend a portion of each day in some productive labor was good for the mental faculties also.

In our profession this careful economy of time, and unremitting industry is necessary, that after the duties of a living practice we may have time for study, without which we can not keep step with the advancements in modes of practice for which our period is remarkable. Any one who fails to keep abreast with this progress will also fail of honorable success.

Many have embraced the profession of dentistry, because of their aversion to labor, as many have done with other professions. But it is a ruinous mistake. If one desires to do nothing comfortably, he should assume as little prominence for his base of operations as possible.

But with these four leading requirements for success in dentistry there must be still others. And next I would rank courtesy or politeness. None of the affected article; but that which springs from a right feeling toward our fellow-beings. Every exercise of kind feelings will certainly increase them, so that what is begun as a duty will soon become a pleasure. Careful regard for a good conscience will produce an increase of genuine politeness. Seek as much to feel kindness as to express it. Cheerfulness is also essential to politeness. You can scarcely be cheerful without being polite. We all know how much more we desire the presence of our cheerful acquaintances, than those who can not avoid gloomy thoughts and reflections. People apply the same rule to the choice of a physician, or dentist, or any one who is to occupy an important or confidential business, or other relation to them.

It is not my purpose in this connection to urge upon your notice all the considerations that might effect your usefulness, as individual hygiene, temperance, restraint of passions, to mind your own business except when charity calls you, to avoid the extremes of avarice, prodigality, or any special plea for each of the Ten Commandments. I believe that what I will call the five cardinal points, that I have endeavored to present, and at their true worth, viz: natural fitness, heartiness in the work, proper training, industry, and considerate treatment of others, will make a man well nigh invincible in the contest for professional success.

